# Modeling Propagation of COVID-19 in the UK

**DOI:** 10.1017/dmp.2020.383

**Published:** 2020-10-13

**Authors:** Babak Jamshidi, Hakim Bekrizadeh, Shahriar Jamshidi Zargaran, Mansour Rezaei

**Affiliations:** Department of Biostatistics, Kermanshah University of Medical Sciences, Kermanshah, Iran; Department of Statistics, Payam-e-Noor University, Iran; Department of Medical Engineering, Tehran University of Medical Sciences, Tehran, Iran; Social Development and Health Promotion Research Center, Kermanshah University of Medical Sciences, Kermanshah, Iran

**Keywords:** COVID-19, modeling, propagation

Coronavirus disease (COVID-19) was first identified in December 2019 in Wuhan, China. From the beginning, this disease has been the subject of various scientific studies. Due to the large amount of data related to the spread of COVID-19 and the high speed of changes, particularly in modeling and forecasting works, it is required to update the predictions and assess the goodness of performance or the accuracy of the models. In this regard, we aim at evaluating the performance of the model introduced by Jamshidi et al.^1^ to describe the first wave of infectious diseases. Since about the propagation of COVID-19 in the UK, until early July 2020, we had encountered the first wave of the disease, and it is possible to examine the performance of the model to describe the trend of the disease up to early July. Therefore, in this letter, we want to evaluate the performance of the model in 2 periods:

- The time studied by Jamshidi et al.^2^ (April 15 to May 30, 2020)
- A 1-month period thereafter (May 31 to July 1)

## PREDICTION VERSUS REALITY

Based on the calculations of Jamshidi et al.,^2^ the following estimated parameters for the model were obtained to describe the daily relative increment of confirmed cases and case fatality rate, respectively:
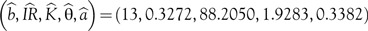


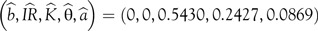



**FIGURE 1:**
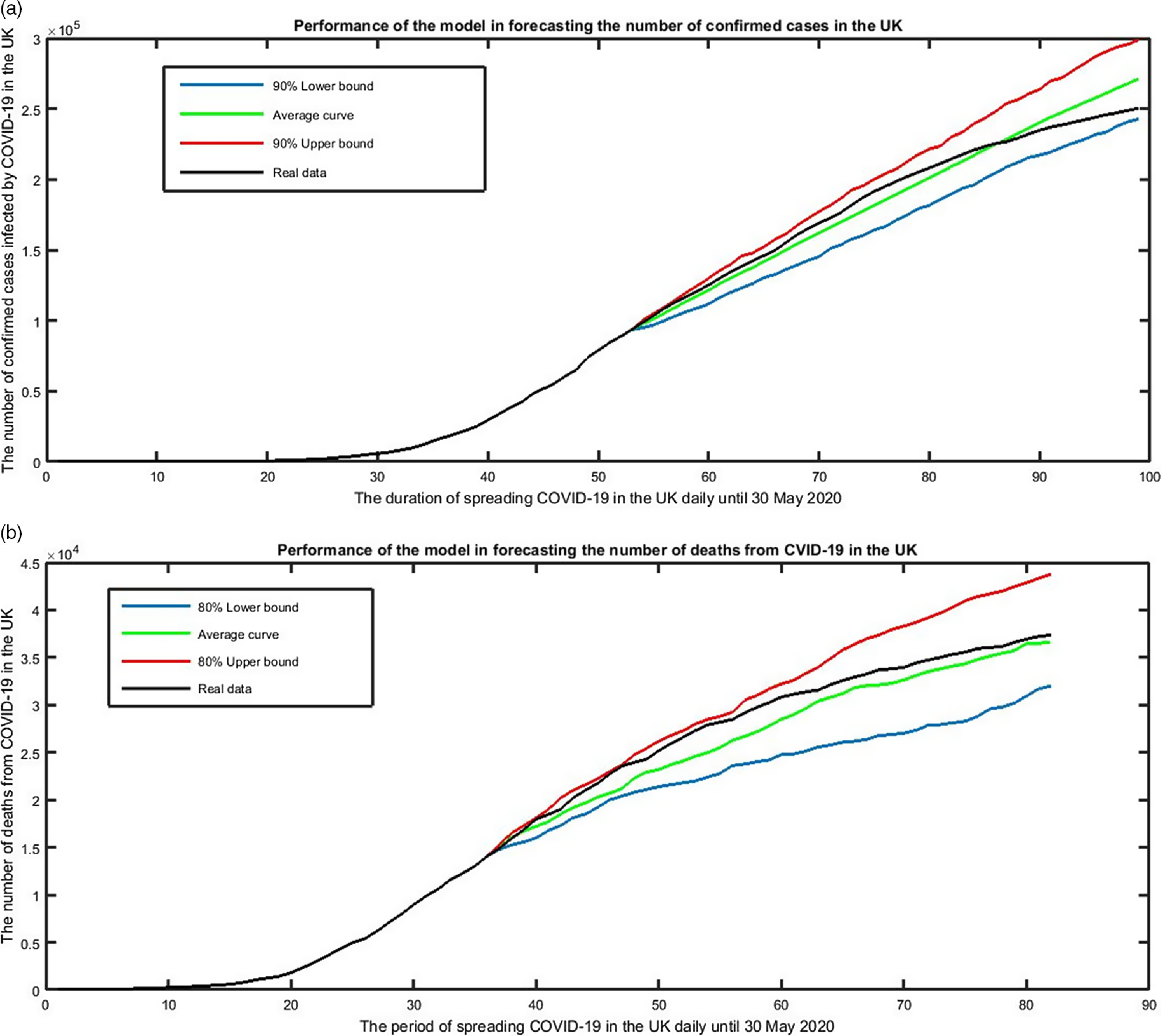
Performance of the Point Estimation and 80% Confidence Interval Based on the Obtained Model in Forecasting the Cumulative Number of Confirmed Cases and Deaths in the UK on May 30, 2020.

The above estimations were based on the time series of the number of confirmed cases and deaths until April 14, 2020. Jamshidi et al.^2^ applied the above models to forecast the propagation of the pandemic in the UK from April 15 to May 30, 2020. Accordingly, they yielded 282 K and 31 K as point estimations and 242–316 K and 28–50 K as 80% confidence intervals for the cumulative number of confirmed cases and deaths on May 30, 2020, respectively. By repeating the simulation, we obtained 273 K and 243–300 K (Figure 1A) and 36 K and 32–43 K (Figure 1B) as predictions. Since, there were 250 347 cases and 37 529 deaths reported in the UK by the mentioned date, and in reality, both of the intervals include the real data, and the relative error of the 2 point estimations are *12% and 9%* and *16% and 5%* for cases and deaths, respectively.

Similarly, by considering the data up to May 30 and calculating the model fit for them, we get the following estimated parameters to describe the daily relative increment of confirmed cases and case fatality rate, respectively:
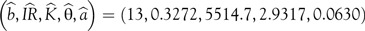


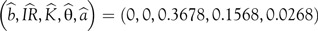



According to these models, we obtained 291 K and 279–304 K (90% confidence interval) as forecasts of the cumulative number of reported cases in the UK on July 1, 2020, which was 284 000. Similarly, the point estimation of 45 K and the 90% confidence interval of 38–53 K were obtained as the forecast of the cumulative number of deaths in the UK on July 1, which was 40 490. Accordingly, both of the intervals include the real data, and the relative errors of the point estimations are less than 3% and 11%, respectively.
